# A Narrative Review of the Many Psychiatric Manifestations of Neurosyphilis: The Great Imitator

**DOI:** 10.7759/cureus.44866

**Published:** 2023-09-07

**Authors:** Baneet Kaur, Deepesh Khanna

**Affiliations:** 1 Department of Medicine, Nova Southeastern University Dr. Kiran C. Patel College Of Osteopathic Medicine, Clearwater, USA; 2 Department of Foundational Sciences, Nova Southeastern University Dr. Kiran C. Patel College of Osteopathic Medicine, Clearwater, USA

**Keywords:** capgras syndrome, dementia, geschwind syndrome, neurosyphilis, personality changes, psychiatric manifestations of neurosyphilis, psychosis, syphilis, tertiary syphilis, treponema pallidum

## Abstract

Neurosyphilis is an infection of the central nervous system caused by the spirochete, *Treponema pallidum*. New syphilis infections have been increasing around the world each year. This disease was much of a concern in the pre-penicillin era, where when left untreated many cases progressed to tertiary syphilis which can commonly manifest as neurosyphilis. Of particular interest, neurosyphilis has been linked to masquerading itself as various psychiatric conditions. This narrative review focuses on exploring psychiatric manifestations of neurosyphilis as well as the importance of screening in psychiatric settings and clinicians maintaining high clinical suspicion of the disease. A systematic search was conducted for published articles from 2003 to 2023 using PubMed, EMBASE, and Google Scholar. A total of 66 articles met the criteria and were used for detailed analysis, where psychiatric manifestations and clinical progression of patients were discussed in detail. Psychiatric manifestations that were explored include dementia, delirium, depression, mania, personality changes, and psychosis. One of the most common manifestations of neurosyphilis appears to be severe neurocognitive impairment. There are also rare psychiatric conditions neurosyphilis mimics that have been described in literature such as Capgras syndrome and Geschwind syndrome. A narrative review of the literature revealed a low level of clinical awareness of neurosyphilis as a possible etiology of various psychiatric disorders. This resulted in delayed or inaccurate diagnosis and consequently delayed initiation of adequate treatment. Considering that many psychiatric manifestations of neurosyphilis are reversible with proper treatment, it is imperative to implement routine screening for syphilis among psychiatric patients.

## Introduction and background

Syphilis, often dubbed the great imitator, is an infection caused by the spirochete *Treponema pallidum* [1]. The World Health Organization projects an estimated 10 to 12 million new syphilis infections around the world each year [1]. Transmission commonly occurs sexually through direct contact with the active infectious lesion, termed a chancre, but may also occur vertically during pregnancy as the spirochete can readily cross the placenta [2]. Syphilis commonly progresses through various clinical stages in its disease course. The disease is classified as primary, secondary, and tertiary syphilis. Neurosyphilis is one such complication of syphilis when left untreated [2].

Neurosyphilis is an infection of the central nervous system. It is worth noting that despite confusion in the literature that often alludes to neurosyphilis as a late complication of syphilis, it can in fact present at any time or in any stage of the disease course [2]. Neurosyphilis early on infects the CSF and meninges. It later involves the brain and spinal cord parenchyma [3]. This disease was much of a concern in the pre-penicillin era, where nearly 30% of patients with syphilis developed tertiary syphilis which can commonly manifest as neurosyphilis [4]. In today’s day and age, it appears that neurosyphilis is far more common in those who are coinfected with HIV as the body turns to a more immunocompromised state and lacks an appropriate response to the infection. The disease burden of neurosyphilis can be severe without treatment [4].

Early neurosyphilis can present with symptomatic meningitis, ocular syphilis, otosyphilis, and/or meningovascular syphilis [5]. Notably, early neurosyphilis may even present as asymptomatic, but treatment is nevertheless recommended to prevent progression to symptomatic disease. Late neurosyphilis commonly presents as general paresis, also termed paretic neurosyphilis or dementia paralytica, which is a progressively dementing illness [5]. Late neurosyphilis may also present as tabes dorsalis, which results in degeneration of the nerve cells in the dorsal columns of the spinal cord that carry sensory information to the brain [5].

The manifestations of general paresis are sometimes described using the acronym PARESIS which stands for the involvement of personality, affect, reflexes, eye, sensorium, intellect, and speech [6]. As a consequence of brain parenchymal invasion by spirochetes, late neurosyphilis can notably mimic various psychiatric conditions - some of which include depression, mania, psychosis, hallucinations, delusions, elation, dementia, and schizophrenia-like illness [6]. The frequency of psychiatric signs and symptoms associated with neurosyphilis ranges from 33% to 86% as reported in the literature [7]. Personality changes, dementia, abnormal behavior, and emotional problems seem to be the most common psychiatric manifestations according to a 2011 retrospective study from China, although depression, psychosis, and mania have also been noted [8].

Neurosyphilis is often not high on clinical suspicion, which results in delayed diagnosis of patients. Zheng et al. in their 2011 study found that neurosyphilis was not suspected in around 36% of patients with paresis-like symptoms, which delayed appropriate diagnosis for 1 to 24 months [8]. Although the venereal disease research laboratory (VDRL) serum test and rapid plasma reagin (RPR) test are screening tests for syphilis, the Centers for Disease Control and Prevention (CDC) outlines two diagnostic categories that must be met for a diagnosis of neurosyphilis [9]. The first category is “confirmed” neurosyphilis where neurosyphilis is present in any stage of syphilis with a reactive CSF-VDRL test [7]. The second category is “presumptive” neurosyphilis where there is a non-reactive CSF-VDRL test but is accompanied by CSF abnormalities such as pleocytosis or elevated protein, and clinical signs and symptoms consistent with syphilis [7]. It is well documented that asymptomatic neurosyphilis exists and presents itself with abnormal CSF findings on lumbar puncture due to the spirochetes invading the CSF [10]. Asymptomatic neurosyphilis can progress to symptomatic disease states and should be treated accordingly to prevent this from occurring [5]. It is, therefore, imperative to reconsider the guidelines for screening for asymptomatic neurosyphilis.

Mainstay treatment continues to be aqueous crystalline penicillin G or procaine penicillin G [10]. Alternate treatment options include ceftriaxone or high-dose doxycycline therapy for those with penicillin allergy [11]. Neurological symptoms may worsen in patients being treated for syphilitic dementia. This can be attributed to the Jarisch-Herxheimer reaction (JHR), which is thought to be an acute inflammatory reaction that is mediated by the rapid lysis of the spirochetes after antibiotic induction [12]. The JHR presents as fevers, rashes, and body aches and usually resolves within 24 hours of administration of the antibiotic [12]. Moreover, *Treponema pallidum* does not always result in persistent infection. Although this is not commonly seen in other bacterial infections of the CSF, *Treponema pallidum* can resolve spontaneously [2].

Misdiagnosis can often complicate and hinder the timely treatment of neurosyphilis. This can be due to neurosyphilis presenting primarily with psychiatric symptoms without other common neurological signs and symptoms. This leads clinicians to pursue diagnoses that are primarily psychiatric in nature such as dementia, psychosis, or mood disorders. This is where screening for neurosyphilis in a psychiatric setting can be of high value as alluded to above. This paper aims to explore the various psychiatric manifestations of neurosyphilis and the value of early screening in psychiatric settings.

## Review

Methodology

A narrative review of neurosyphilis and its many psychiatric sequelae was performed using online databases such as PubMed, Google Scholar, EMBASE, and CDC's website. The key search terms were as follows: (neurosyphilis psychosis) OR (neurosyphilis psychiatric) OR (neurosyphilis psychiatric complication) OR (neurosyphilis psychotic) OR (neurosyphilis psychiatric sequelae). The articles selected pertained to neurosyphilis and its many psychiatric manifestations. A screening of the literature was carried out based on inclusion criteria as follows: psychiatric manifestations in neurosyphilis-positive patients, human subjects, English language, and a date range from 2003 to 2023. Case reports, clinical trials, observational studies, randomized control trials, clinical studies, meta-analyses, and systemic reviews were all included in the literature search. Articles that did not mention psychiatric manifestations as a result of neurosyphilis were excluded. A Preferred Reporting Items for Systematic Reviews and Meta-Analyses (PRISMA) diagram was created to illustrate the selection and screening process of these articles. A total of 66 articles met all inclusion criteria and were included for the purposes of the literature review.

**Figure 1:**
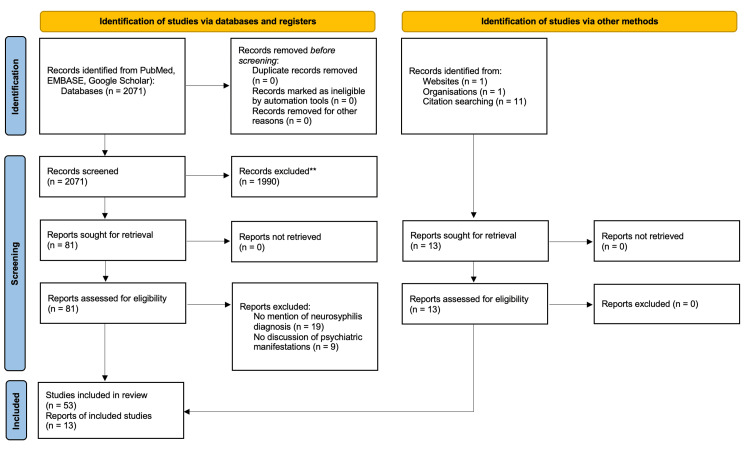
Study selection PRISMA flow diagram

Discussion

Neurocognitive Impairment

One of the most common manifestations of neurosyphilis appears to be severe neurocognitive impairment. This has been extensively described in the literature. A significant number of patients with neurosyphilis present with cognitive impairment and delirium. In one case study of 52 neurosyphilis patients, 34% presented with cognitive impairment and 25% with signs of delirium [13]. The delirium can often progress to dementia, and this presents with an insidious course of dementia that “leads to progressive global deterioration in intellectual functioning” [13]. Rapidly progressive dementia is commonly seen in the meningovascular stage as there are signs of vascular dementia-like symptoms through the ischemic strokes defined in this stage of the disease or due to hydrocephalic changes from the blockage of CSF flow [14]. Neurosyphilis should be considered as a treatable cause of forms of dementia as treating the underlying infectious process can lead to the improvement of clinical symptoms. Delayed diagnosis and treatment can, however, lead to minimal or no improvement of symptoms as it leads to advanced stages of neurosyphilis.

In a 2008 retrospective study by Luo et al., they reviewed five patients diagnosed with general paresis. Four cases exhibited cognitive impairment, two also had urinary incontinence, and two had psychosis. The average disease progression was around 20 months. There was marked cerebral atrophy in all five cases, with the RPR test testing being positive in all cases as well. As mentioned above, the RPR test is an appropriate initial screening test and is reported as a quantitative titer. This titer can be followed to track disease progression or resolution. Subsequent examination of the CSF showed abnormal findings in all patients. All patients were treated with high-dose penicillin, which was followed by a marked improvement in clinical symptoms in four patients. In one of the cases, the symptoms were too advanced as there was a prolonged time from symptomology presentation, diagnosis, and appropriate treatment. For instance, in one of the five cases, the patient’s CSF RPR decreased from 1:16 to 1:8, and the mini-mental state examination (MMSE) increased from 14 to 26 after two weeks of treatment [15]. This illustrates the importance of keeping neurosyphilis high on the differential list for those who present with abrupt or unexplained neurocognitive decline, as appropriate treatment can improve the neurocognitive symptoms. It was illustrated through four of the cases that appropriately treating the underlying neurosyphilis led to remarkable improvement of cognitive symptoms as evidenced through the MMSE in one of the patients [15]. Unfortunately, in the case of delayed diagnosis and treatment, it can lead to little to no improvement of symptoms as evidenced in one of the patients in this study.

In another case, Caroppo et al. described the findings of a 59-year-old man who presented with clinical symptoms of a behavioral variant of frontotemporal dementia [16]. The patient developed psychiatric disturbances with verbal aggressiveness over the course of a few months, which was followed by cognitive and behavioral disorders. There was marked atrophy and hypometabolism in the frontotemporal regions. Screening exams for syphilis turned out to be positive in subsequent testing, and there were abnormal CSF findings which pointed to neurosyphilis as the diagnosis. With antibiotic treatment, the patient's behavioral symptoms along with cognitive impairments showed marked improvement, confirming neurosyphilis as the cause of the psychiatric disturbance [16]. This case illustrates that neurosyphilis can mimic frontotemporal dementia and should, therefore, be kept high on the differential diagnosis as treatment can halt disease progression and lead to remarkable improvement in the disease course.

Moreover, Toptan et al. in a 2015 case report described the findings of a patient with neurosyphilis presenting with progressive cognitive decline and intractable behavioral and psychiatric issues. They describe a 40-year-old male patient with amnesia, personality changes, aggressive behaviors, hallucinations, and illusions for one year. His symptoms started three years prior with unwillingness to move and loss of appetite being some of the first described symptoms. Further testing showed normal blood analyses of complete blood count and hepatic, renal, and electrolyte levels. Markers of vasculitis were negative. VDRL and RPR tests were, however, positive [5]. Cranial MRI showed cerebral and cerebellar atrophy. Lumbar puncture found abnormal CSF findings. A diagnosis of neurosyphilis was made upon ruling out differentials such as primary dementia, vascular dementia, and space-occupying mass lesions in the brain, and appropriate treatment with penicillin was initiated. Unfortunately, the patient did not show improvement in cognitive functions as it was an evident case of advanced neurosyphilis with three years passing between the first sign of symptoms to the appropriate diagnosis [5].

Personality Changes

Neurosyphilis is often associated with personality changes that include aggressiveness and paranoia. According to Lin et al., personality changes were the second most common psychiatric sequelae in a case study of 52 patients, where 53.9% of patients had developed some sort of personality disorder as a manifestation of neurosyphilis [13]. Frontotemporal atrophy can be attributed to personality and behavior changes, which has been seen on imaging in cases of neurosyphilis presenting with personality changes [17]. Other explanations include increases in cerebral blood flow that may reflect an inflammatory state due to the infectious process of neurosyphilis [18].

In a case report by Ide et al., a 31-year-old woman presented with emotional and personality changes that were attributed to a diagnosis of neurosyphilis [18]. Notably on imaging, MRI was unremarkable but N-isopropyl-p-(123I)-iodoamphetamine single photon emission computed tomography (123I-IMP SPECT) showed an increase in cerebral blood flow [18]. The increase in cerebral blood flow is postulated by the authors to be due to the inflammatory state of neurosyphilis [18]. Upon treatment with penicillin, not only did the clinical symptoms that she presented improve, but the increase in cerebral blood flow disappeared as well. The disappearance of the increased cerebral blood flow then suggests a decrease in the inflammatory state caused by neurosyphilis. The authors suggest that effective treatment can be marked through SPECT imaging in these cases as the decrease in blood flow can be quantitively measured to ensure the treatment regimen is effective.

The importance of correct diagnosis and prompt treatment is illustrated through the case of an elderly woman who progressively developed personality changes and paranoid delusions in addition to cognitive impairment over a period of six months [19]. Although Alzheimer’s disease was suspected initially, it was ultimately found that she had neurosyphilis. Antibiotic treatment resulted in the attenuation of psychotic symptoms. Had the initial suspected diagnosis of Alzheimer’s stuck, it may have been detrimental to the patient’s care plan.

Mood Disorders

Mood disorders such as mania and depression have been described as a psychiatric manifestation of neurosyphilis. There are a limited number of case reports on mood disorders and neurosyphilis specifically, however, of those depressive illness appears to be the most common [20]. Although there is no exactly understood mechanism as to why these mood disorders precipitate as a result of neurosyphilis, what is understood is that symptoms of mood disorders in neurosyphilis are commonly atypical and fail to meet DSM-5 diagnostic criteria [21]. Much of the explanation of psychiatric manifestations stands on the fact that there is extensively damaged brain parenchyma in cortical regions [13].

In a case report by Crozatti et al., a 48-year-old male presented with a two-week history of malaise and headache which progressed to a depressed mood and disorientation a week later [7]. The patient attempted suicide and there was no history of previous psychiatric disease or episodes of this nature. CSF fluid was examined, a diagnosis of herpes simplex virus encephalitis was suspected, and intravenous acyclovir was initiated. Seven days into the therapy, no clinical improvement was noted. A VDRL test was requested and subsequently resulted positive which led to the diagnosis of neurosyphilis. Treatment protocol with penicillin was started. Thirty hours into antibiotic therapy, there was evident clinical improvement: from the Glasgow Coma Scale of 10 on presentation rising to 15 after appropriate treatment. At the end of the therapy, the patient returned to normal behavior [7]. This case illustrates again the dangers of misdiagnosis and poor clinical outcomes contrasted with appropriate diagnosis and treatment initiation which can result in complete reversal of symptoms. In another case of mood disorders, a 46-year-old male with no psychiatric history presented with abrupt signs of mania [22]. Although he was initially diagnosed with bipolar disorder with psychotic features, by day three of admission, neurosyphilis was confirmed and treated accordingly. After two weeks of treatment, most of the patient's symptoms had subsided [22].

Psychosis

Psychosis as the first manifestation of neurosyphilis has only been reported in a limited number of cases in the literature [23]. Although many of these neuropsychiatric sequelae overlap and co-exist, psychosis can present as a stand-alone psychiatric manifestation and is often an ominous symptom of neurosyphilis [24]. These patients who present with psychosis as the first manifestation are often misdiagnosed with late-onset schizophrenia [23]. This complicates treatment and prolongs the disease course in these individuals.

Wahab et al. described the case of a male patient in his 40s who was brought into the hospital by police due to aggressive and bizarre behavior [25]. He had signs of irrelevant speech and restricted affect. Moreover, he was disoriented by time, place, and person. A provisional diagnosis of schizophrenia with possible delirium was made. The patient displayed a poor response to antipsychotics. Subsequent testing found HIV to be negative, but the VDRL titer was 1:32 [25]. This changed the diagnosis to neurosyphilis, and appropriate treatment with antibiotics was commenced. Some of the patient’s symptomology improved, notably the memory deficit, but the irrelevant speech remained [25]. This highlights a case where misdiagnosis can lead to the treatment of refractory psychosis and poor resolution of symptoms due to late diagnosis and treatment for neurosyphilis.

In another case, Moolla and Abdul described a case of a 52-year-old male who was brought into the hospital by police and held involuntarily [26]. His symptoms included aggression toward his family, and collateral information collected from the family indicated a sudden change in his personality and blunted emotional response over the past few weeks. Upon questioning, the patient repeatedly stated an ability to communicate with God, which suggested a delusional state. The clinical examination noted no signs of trauma or substance use. An antipsychotic was initiated, but the delusional state and mild aggression persisted. Further testing allowed an appropriate diagnosis of neurosyphilis to be made. Treatment with penicillin led to complete resolution of symptoms and the patient was discharged [26]. This again makes it evident that even in cases of psychosis, timely treatment with penicillin following a correct diagnosis can lead to a complete reversal of clinical symptoms. This is quite remarkable considering these patients would otherwise be labeled as treatment refractory cases of psychosis.

Other

There are also rare psychiatric conditions that have been described in the literature. Capgras syndrome is a specific delusion where a patient believes that a family member has been replaced by an imposter who is identical [27]. There is one such case, which based on the search is the first to be reported in the literature, described by Chang and Tsai, where Capgras syndrome was an initial presentation of neurosyphilis. It is the case of a 52-year-old who two months prior to admission developed delusions that her family members were deceased and replaced by individuals pretending to be her family [27]. An initial diagnosis of late-onset schizophrenia was made, which was deemed inappropriate due to disease progression. A subsequent positive RPR and abnormal CSF findings led to the diagnosis of neurosyphilis.

In another case, Toffanin et al. described the case of a 34-year-old woman with neurosyphilis limbic encephalitis who presented with Geschwind syndrome, which is characterized by hyperreligiosity and hypergraphia and mood disorder [28]. This was reversible with acoustic pulse therapy treatment with antibiotics. These rare psychiatric manifestations again illustrate the need to screen for neurosyphilis in psychiatric settings. It is obvious that appropriate treatment for neurosyphilis can reverse symptomology leading to improved patient outcomes.

Table 1 summarizes the findings of the studies included in this review.

**Table 1:** Summary of the one retrospective case series and 10 case reports describing the various psychiatric manifestations of neurosyphilis in a total of 15 patients RPR: rapid plasma reagin, VDRL: venereal disease research laboratory, LP: lumbar puncture, CSF: cerebrospinal fluid, MMSE: mini-mental state examination

Authors and year	Country	Study design	Demographics/presentation	Findings
Toptan et al. (2015)[5]	Turkey	Case report (n=1)	A 40-year-old male who presents with progressive cognitive decline accompanied by behavioral and psychiatric issues for three years.	The patient over time showed symptoms of amnesia, personality changes, aggressive behavior, hallucinations, and illusions. RPR and VDRL, along with LP findings, proved the diagnosis to be neurosyphilis. Antibiotic treatment was unfortunately not successful, demonstrating advanced neurosyphilis.
Crozatti et al. (2015)[7]	Brazil	Case report (n=1)	A 48-year-old male presents with a two-week history of malaise and headache that quickly progressed to depressed mood and disorientation one week later.	Although herpes encephalitis was suspected, a positive VDRL led to the diagnosis of neurosyphilis. Thirty hours into antibiotic treatment showed marked clinical improvement, with a return to baseline upon completion of treatment.
Luo et al. (2008)[15]	China	Retrospective case series (n=5)	Five patients were diagnosed with neurosyphilis of various ages and sex.	Four out of the five cases demonstrated cognitive impairment, where two also had urinary incontinence and another two also had psychosis. RPR testing was positive in all cases and LP showed abnormal CSF findings in all five patients as well. The patients were then treated with penicillin which showed marked clinical improvement in four out of the five patients. This was demonstrated in one of the patients with the MMSE improving from 14 to 26 after two weeks of treatment. One of the cases showed little improvement, suggesting the disease had progressed to advanced neurosyphilis due to a lack of timely diagnosis and treatment.
Caroppo et al. (2022)[16]	Italy	Case report (n=1)	A 59-year-old man who presents with complaints of frontotemporal dementia.	The patient had psychiatric disturbances with verbal aggressiveness. Syphilis testing and LP demonstrated a diagnosis of neurosyphilis. With antibiotic treatment, there was considerable improvement in the patient’s cognitive impairments.
Ide et al. (2004)[18]	Japan	Case report (n=1)	A 31-year-old woman presents with emotional and personality changes.	The changes were attributed to neurosyphilis, which showed improvement upon penicillin treatment. The authors describe 123I-IMP-SPECT findings in this patient which they postulate can be used to quantitatively measure treatment success in neurosyphilis.
Güler and Leyhe (2011)[19]	Germany	Case report (n=1)	An elderly woman who progressively displayed personality changes and paranoid delusions, in addition to cognitive impairment over six months.	A diagnosis of Alzheimer’s was suspected, but further testing proved neurosyphilis to be the correct diagnosis. Prompt treatment with antibiotics led to a decrease in psychotic symptoms.
Seo et al. (2018)[22]	South Korea	Case report (n=1)	A 46-year-old male who abruptly presents with signs of mania.	An initial diagnosis of bipolar disorder with psychotic features was quickly turned down with testing proving neurosyphilis to be the correct diagnosis. Two weeks of treatment resulted in great clinical improvement with most of the patient’s symptoms subsiding.
Wahab et al. (2013)[25]	Malaysia	Case report (n=1)	A male in his late 40s brought to the hospital by police presents with aggressive and bizarre behavior.	The patient was disoriented to person, place, and time, in addition to signs of irrelevant speech and restricted affect. A diagnosis of schizophrenia was made, but treatment with antipsychotics was unsuccessful. Subsequent testing proved neurosyphilis to be the appropriate diagnosis. Treatment with antibiotics showed clinical improvement.
Moolla and Abdul (2016)[26]	South Africa	Case report (n=1)	A 52-year-old male brought into the hospital by police presents with aggression, a sudden change in personality, and a blunted emotional response over the course of a few weeks.	The patient repeatedly stated an ability to communicate with God. An antipsychotic was ineffective in resolving the delusional state and aggression. A diagnosis of neurosyphilis was made, and treatment with penicillin led to complete resolution of symptoms.
Chang and Tsai (2015)[27]	Taiwan	Case report (n=1)	A 52-year-old male presents with a belief that his family members are imposters, consistent with Capgras syndrome which develops in many schizophrenic patients.	A diagnosis of late-stage schizophrenia was made, but subsequent testing revealed neurosyphilis, illustrating that Capgras-like syndrome can be the initial presentation of neurosyphilis.
Toffanin et al. (2019)[28]	Italy	Case report (n=1)	A 34-year-old woman presents with Geschwind syndrome, which was later confirmed to be due to neurosyphilis.	This case demonstrates odd initial presentations of neurosyphilis.

## Conclusions

With its ability to masquerade and guise itself as multiple psychiatric conditions, neurosyphilis can be an ominous condition clinicians must be prepared to treat. These are often reversible with accurate diagnosis leading to timely treatment. Delayed treatment, however, can result in irreversible conditions. Healthcare providers should keep neurosyphilis high on the differential list when faced with patients who either are non-responders to typical treatment of these conditions or patients who have unusual presentations. It can often be hard to distinguish neurosyphilis from primary psychiatric disorders, especially in the setting of unusual and atypical presentations. This is why it is imperative we make it common practice and a standard to screen psychiatric patients for syphilis. Moreover, retrieving a thorough history from the patient and collaterals can provide information on potential current or past risky behavior that can put the patient at a higher risk of being infected with syphilis. These measures can lead clinicians to accurately and promptly diagnose neurosyphilis, resulting in a little delay in treatment. However, the study did not compare the outcomes of patients with neurosyphilis who received timely treatment versus those who received delayed treatment. This may limit the generalizability and applicability of the results. Furthermore, the study did not control for confounding factors that may influence the presentation and response to treatment of neurosyphilis, such as comorbidities, medications, substance use, and sociodemographic variables.
