# Correction: A Narrative Review of the Many Psychiatric Manifestations of Neurosyphilis: The Great Imitator

**DOI:** 10.7759/cureus.c362

**Published:** 2025-10-15

**Authors:** Baneet Kaur, Deepesh Khanna

**Affiliations:** 1 Department of Medicine, Nova Southeastern University Dr. Kiran C. Patel College Of Osteopathic Medicine, Clearwater, USA; 2 Department of Foundational Sciences, Nova Southeastern University Dr. Kiran C. Patel College of Osteopathic Medicine, Clearwater, USA

This article has been corrected to fix the following typo in the PRISMA diagram: 'Records screened' corrected from 2,083 to 2,071.

